# Evaluation of partial volume effect correction methods for brain positron emission tomography: Quantification and reproducibility

**DOI:** 10.4103/0971-6203.35723

**Published:** 2007

**Authors:** Merisaari Harri, Teras Mika, Hirvonen Jussi, Olli S. Nevalainen, Hietala Jarmo

**Affiliations:** * Turku PET Centre, University of Turku / Turku University Central Hospital, Turku, Finland; ^ Department of Information Technology, University of Turku, Turku, Finland; § Department of Psychiatry, University of Turku, Turku, Finland

**Keywords:** Brain imaging, partial volume effects, positron emission tomography

## Abstract

Quantitative accuracy of positron emission tomography (PET) is decreased by the partial volume effect (PVE). The PVE correction (PVC) methods proposed by Alfano *et al.*, Rousset *et al.*, Müller-Gärtner *et al.* and Meltzer *et al.* were evaluated in the present study to obtain guidelines for selecting among them. For accuracy evaluation, the Hoffman brain phantom was scanned with three PETs of differing spatial resolution in order to measure the effect of PVC on radioactivity distribution. Test-retest data consisting of duplicate dynamic emission recordings of the dopamine D2-receptor ligand [^11^C] raclopride obtained in eight healthy control subjects were used to test the correction effect in different regions of interest. The PVC method proposed by Alfano *et al.* gave the best quantification accuracy in the brain gray matter region. When the effect of PVC on reliability was tested with human data, the method of Meltzer *et al.* proved to be the most reliable. The method by Alfano *et al.* may be better for group comparison studies and the method by Meltzer *et al.* for intra-subject drug-effect studies.

Positron emission tomography (PET) is a medical imaging method that records the simultaneous emission of gamma rays from the point of positron-electron annihilation in living tissue. Neuroreceptors and cerebrometabolic pathways can be targeted with specific tracers which bear positron-emitting isotopes in their chemical structure. Unfortunately, despite the good quantitative capabilities of PETs, the PET imaging technique suffers from relatively poor spatial resolution, which impairs the spatial localization of the radioactivity signals and also results in the so-called partial volume effects (PVE). As a result of PVE, the quantitative accuracy of PET images is reduced. The PVE smoothes PET images so that some of the radioactivity from regions of higher concentration is mis-attributed to adjacent regions of lower activity. In brain imaging with tracers of high radioactivity in brain gray matter (GM), PVE yields lower radio activities than their real values for GM structures and higher radioactivity values for the brain white matter (WM). As a result of this, the radioactivity concentration values are differently variated in different regions of interest (ROI) of the brain.

The main correlating element for PVE is the size of the image resolution, which is mainly determined by the crystal size, the ring diameter of the tomograph and the travel distance of the positron. The travel distance before annihilation degrades the resolution, and it correlates with the kinetic energy of the positron of the used tracer. Other contributing factors include the size and shape of the radioactivity source and the signal-to-noise ratio of the PET image. In addition to the physical causes of PVE, also the computational methods that are used in order to reconstruct the PET image can induce PVE in the resulting image. These include histogramming and reconstruction from coincidence raw data to sinogram data, sinogram data reconstruction to PET images and image post-filtering after reconstruction of the PET images.

Various methods for partial volume effect correction (PVC) have been proposed. The method reported by Meltzer *et al.*[1] corrects the radioactivity values of the conjunction of the WM and GM regions. The methods of Müller-Gärtner[2] and Alfano *et al.*[3] process the WM and GM regions separately and use an estimate of the true WM mean radioactivity when correcting the radioactivity values of the GM region. Modifications of these two methods have also been proposed.[4] Rousset *et al.*[5] have proposed a method for correcting the mean activity values of ROIs. Most methods mentioned above have been further refined in subsequent years,[6,7] and the robustness of the PVC methods against errors in their processing steps such as MRI-PET co-registration and MRI segmentation has also been considered in several studies.[8–11] The method of Meltzer *et al.* has been reported to be robust to errors in preprocessing and in homogeneities in the PET image.[10]

It is notable that the methods amplify the existing noise.[12] An increase of variance in the time-activity curves has been reported when using the method by Rousset *et al.*[13] If the noise is amplified too much, the usefulness of recovering the regional mean radioactivity values decreases. Therefore, both the effect of PVC on noise and on the accuracy of the mean values of ROIs should be evaluated. Further, when dispersion in image voxels is high, it is difficult to conclude whether small differences in images or regions originate from real differences in the studied objects, or if they are caused by the data-collecting and reconstruction processes.[14] The PET method is typically used in group comparison studies and intra-subject drug effect studies. The application of a PVC method is sensible if it is capable of revealing the true differences between the groups and between the scans regardless of the added noise.

In the present study, testing with a phantom object was done using three different tomographs (GE Advance, GE DSTE and Siemens HRRT) to reveal differences in the noise amplification between the PVC methods proposed by Alfano *et al.,* Rousset *et al.,* Müller-Gärtner *et al.* and Meltzer *et al.* In total, eight methods were evaluated, as three different WM radioactivity estimation methods proposed by Alfano *et al.,* Rousset *et al.* and Müller-Gärtner *et al.* were combined with the methods by Alfano *et al.* and Müller-Gärtner *et al.* In addition, the binding potential values with eight patients scanned on two separate occasions with the dopamine D2 receptor ligand [^11^C] raclopride were analyzed with four of the methods in order to reveal the reproducibility and reliability characteristics of the evaluated PVC methods. Real differences between scans are assumed to be minimal in this test-retest material.

## Materials and Methods

Human PET studies lack explicit information on the true radioactivity levels and their distributions. Other means are therefore needed to evaluate the correction methods. Computer simulation is one way to model the imaging process,[15] but this is not easy due to the analog radioactivity signal and due to the complicated behavior of the PET tomograph. While 3D computer simulations allow the definition of arbitrary radioactivity sources with known variable noise levels as input, they may lack the realism of the real scanning situation. They can also require huge amounts of computational resources. Alternatively, phantom objects consisting of fluid-filled chambers can be used. For a phantom object, the radioactivity concentration and distribution are accurately known. Phantoms allow the use of a real tomograph, but the anatomy still differs from that of a real brain. Our approach is to use both phantom and real human studies in the evaluation of PVC methods. This is done in order to obtain more reliable results than would be achieved either with real human subjects or with a phantom alone. Results of real human PET studies also show whether the reproducibility and reliability of the corrected values have decreased between scans. With phantom tests, we can confirm that the results of human studies are not affected by systematic errors. The robustness of correction methods against errors in the structural image segmentation and co-registration to the PET image were not tested here, because such an evaluation has already been done elsewhere.[11,16]

For accuracy evaluation, the Hoffman brain phantom filled with [^18^F] FDG was scanned with three different PET tomographs when evaluating the effect of the PVC on the radioactivity distribution in the case where the actual radioactivity level is known. The test-retest data set consisted of dynamic emission studies of eight healthy human volunteers. Each study consisted of two injections of [^11^C] raclopride with a time separation of at least 2 h. The half life of ^11^C is 20.3 min. All subjects gave informed written consent for their participation in these studies, which were approved by the Ethical Committee of the local health district. The evaluated PVC methods use additional structural information from the imaged object to find the boundaries of the regions to be corrected. For this, computerized tomography (CT) images were used with the phantom object and magnetic resonance imaging (MRI) images with the human subjects. The segmentation of the phantom CT image was performed semiautomatically. The MRI images were segmented with an automatic method that uses predefined probability maps for the segments. PET to MRI co-registration and MRI segmentation were done with SPM2 software (UCL Institute of Neurology, UK, http://www.fil.ion.ucl.ac.uk/spm/software/spm2).

The software used for PVC and the SPM2 were used without GUI to exclude all user interaction. This reduces the time and computational resources needed and makes it possible to run the tests as batch jobs. All images used in the evaluation were converted to Analyze 7.5 image file format before correction.

### Correction methods for PVE

The PVE is generally modeled as a convolution of the real radioactivity values with the point spread function (PSF) and some additional noise:

$$I_{PET,obs}=I_{PET,true}⊗I_{PSF}+noise;\;\mathrm{(2-1)}$$

Here *I_PET,obs_* stands for the radioactivity values of the acquired image including PVE and noise, *I_PET,true_* is the true radioactivity of the object and *I_PSF_* is a PSF kernel. The PSF values are measured by taking a PET image of a point-shaped radioactivity source in air to derive the distribution of the signal in the transaxial and axial directions. The width at one half of the maximal peak height (referred to as FWHM, full width at half maximum) is used as an estimate of the PSF. Another interpretation of FWHM is that it gives the smallest distance between two resolved points such that they can still be distinguished from each other. In this paper, a constant FWHM value for each tomograph is used across transaxial and axial directions for PSF. This is used at the expense of loss of accuracy, but it saves computational resources.[16] The same approach is used for all PVC methods under evaluation, and therefore it is not expected to bias the results.

Formula (2-1) cannot be solved with deconvolution methods directly, because of the contribution of noise term. However, several solutions have been proposed for this problem. In the present work, we focus on PVC methods that are applied to the reconstructed image. These methods can be divided into region-based methods that calculate the corrected mean radioactivity values of certain ROIs and voxel-based correction methods that produce corrected image data. The region-based method originally proposed by Rousset *et al.* (called R-PVC in this paper) divides the image into segments, for which the corrected mean values are calculated by solving a system of linear equations. The voxel-based methods by Meltzer *et al.,* Müller-Gärtner *et al.* and Alfano *et al.* (referred to as M-PVC, MG-PVC and A-PVC respectively in this paper) create a simulated image of the radioactivity distribution after the modeled PVE. The image is then used as a divisor for the voxel values of the region to be corrected. The effect of the PVC on the reproducibility of PET imaging has so far been evaluated only for the M-PVC and MG-PVC methods, with the less sophisticated M-PVC appearing to give better results.[16] However, this method is known to be less accurate compared to the MG-PVC method, which can be seen as a more general solution to the PVC problem.

### Region-based method: R-PVC

In the R-PVC method, the mean radioactivity values *I_j_* (*j = 1, …, N*) for the selected ROIs are modeled by a set of linear equations:

(∫ROI1RSF1(r)dr,…, ∫ROI1RSFN(r)dr∫ROI2RSF1(r)dr,…, ∫ROI2RSFN(r)dr:∫ROI2RSF1(r)dr,…, ∫ROINRSFN(r)dr)(I1I2:IN)=(P1ET obs,ROI1P1ET obs,ROI2:P1ET obs,ROIN)(2-2)

where *I_PET obs, ROI N_* is the observed mean radioactivity of the *N^th^* ROI. This radioactivity is described as a sum which includes the true mean values of all ROIs multiplied by the radioactivity distribution functions RSF. The distribution functions are defined as ROI binary mask images convoluted by the PSF of a particular tomograph (*X_ROI N_ ⊗ I_PSF_*). The observed mean radioactivity concentration in the ROIs is therefore obtained as a sum of the contributions of all defined ROIs to the current ROI area. In the above set of equations, all RSFs and the observed values are known, which makes it possible to calculate the true mean radioactivity values *I_N_*. Furthermore, the values are assumed to be homogenous inside the selected ROIs.

### Voxel based methods: M-PVC, MG-PVC and A-PVC

The voxel-based methods that are evaluated here are based upon a set of assumptions about the PET image under study. In M-PVC, the observed PET image radioactivity concentration *I_PET obs_* is formulated as:

$$I_{PET\;obs}=I_{PET\;act,GM+WM}x\left(X_{GM+WM}⊗I_{PSF}\right);\;\mathrm{(2-3)}$$

where *X_GM + WM_* refers to a binary mask of the WM and GM segments as determined from an MRI image. The segments are convoluted by the tomograph point spread function *I_PSF._* Finally, the true radioactivity concentration at the conjunction of GM and WM regions *I_PET act,GM + WM_* is multiplied by the convoluted mask to produce the observed PET image. In this method, the considerable radioactivity in the observed PET image is assumed to originate only from GM and WM regions. Therefore, the equation can be rewritten as:

$$I_{PET\;act,\;GM+WM}=I_{PET\;obs,\;GM+WM}/\left({\text{X}}_{GM+EM}⊗I_{PSF}\right);\;\mathrm{(2-4)}$$

This method produces a PVE-corrected image containing the composition of GM and WM, with the assumption that the radioactivity concentration is uniformly distributed in these areas.

When ROIs lie mainly within the GM region, it is possible to apply the correction method to that area, assuming that radioactivity is uniformly distributed in the GM and WM regions and that the mean radioactivity of WM is known. In MG-PVC, the observed radioactivity distribution *I_PET obs_* is modeled as a sum of the true GM and WM radioactivity distributions:

$$I_{PET\;obs}=I_{PET\;act,GM}\text{ x}\left({\text{X}}_{GM}⊗I_{PSF}\right)+C_{WM}\text{x}\left({\text{X}}_{WM}⊗I_{PSF}\right);\;\mathrm{(2-5)}$$

$$I_{\text{PET }act,GM}=\;\left[I_{\text{PET }obs}-C_{\text{WM}}\times \left(X_{\text{WM}}⊗I_{PSF}\right)\right]/\left(X_{GM}⊗I_{PSF}\right);\;\mathrm{(2-6)}$$

where *C_WM_* is the estimated mean of the WM radioactivity concentration, *X_WM_* and *X_GM_* are binary masks of the WM and GM segments acquired from the corresponding MRI image and *I_PSF_* is the tomograph PSF. In this method, the correction specializes to the GM area and the corrected mean WM radioactivity concentration is used as a starting point for the algorithm.

The A-PVC method models the observed intensity *I_PET obs_* (*p*) of point *p* by the formula:

$$I_{PET\;obs}\left(p\right)=\int_{volume}C\left(S\right)\;\times \;g\left(p,\;s\right)\;ds\text{ + }noise\text{.}\;\mathrm{(2-7)}$$

The integration is performed over the affecting region (volume) to *p*; *C*(*s*) is the true radioactivity concentration at point *s* and *g*(*p*, *s*) is the probability that *s* contributes to the radioactivity of *p*. Here *g*(*p*, *s*) can be acquired from the tomograph PSF as in M-PVC and MG-PVC. For points in the GM area, the formula becomes:

$$I_{\text{PET }act,GM}\left(p\right)=\int_{volume}I_{\text{PET }obs,GM}\left(S\right)\;\times \;g\left(p,\;s\right)\;ds\text{ + }noise,\;\mathrm{(2-8)}$$

where

$$g\left(p,\;s\right)={\text{X}}_{GM}\left(S\right)\;\times \;g\left(p,\;s\right)/\int_{volume}{\text{X}}_{GM}\left(s\right)\;\times g\left(p,\;s\right)\;ds\text{,}\;\mathrm{(2-9)}$$

which gives the probability that the radioactivity at point *p* originates from point *s*. In (2-8) the observed GM radioactivity is obtained by first subtracting WM radioactivity from the PET image, as in MG-PVC. The assumption about the mean value of WM is the same as with MG-PVC. An essential difference from MG-PVC is that the corrected value of each point *p* is calculated using the observed values from the affecting region (see formulae (2-8) and (2-9)) and not only from a single point as in MG-PVC (see formula (2-6)).

### Summary of correction methods

The above-mentioned assumptions made for the correction methods should be approximately correct for obtaining reliable results inside the GM and WM regions in real applications. Inhomogeneities inside the GM and WM mean that PVE exists also inside them. This does not affect the mean radioactivity concentration, but it changes the values for smaller ROIs inside them. The A-PVC method can be seen as a compromise between region-based and voxel-based methods; and it is assumed to be more accurate than the other voxel-based methods reviewed here, but it is still based upon the same assumptions about the PET images.

### Principles of the WM estimation

The mean radioactivity concentration of the WM affects the MG-PVC and A-PVC methods, and therefore it should be estimated as well as possible. An increase in the WM mean value produces decreased values of the PVE-corrected radioactivity concentrations. In the central slices (CS) method for WM estimation, some transaxial slices from the central area of the brain are selected and the WM segments are then eroded in slices to identify the part of the WM region that is not affected by spillover from the GM. Another way of doing the estimation is to use the method of Rousset *et al.,* reviewed above (referred to as R-WME). Third, in the method proposed by Alfano *et al.*[3] (referred to as A-WME), an image calculated from convoluted mask images with the formula (*X_GM_* ⊗ *I_PSF_*)/((*X_GM_* ⊗ *_PSF_*)+(*X_WM_* ⊗ *I_PSF_*)) is used. In the locations where the values of the image are high, the PET radioactivity values contain little of the true radioactivity from the WM region. Conversely, low values in the image indicate that PET radioactivity values in these locations consist mainly of the true radioactivity from the WM region. In A-WME, the PET radioactivity values are projected against the convoluted mask image. A regression line is then fitted to characterize PET radioactivity values and the probabilities that the radioactivity value originates from the WM region. Finally, the WM estimation is made from an interception of this line.

### Instrumentation

The GE Advance (GE, Milwaukee, WI), PET-CT (DSTE, GE, Milwaukee, WI) and HRRT (CPS Inc., Knoxville, TN) PET tomographs were used for the phantom tests. All three tomographs were operated in the 3D acquisition mode and the images reconstructed with the iterative reconstruction method (OSEM or its variant). All data were dead-time, decay, scatter and attenuation corrected with scanner-specific procedures. The PET input images for the test-retest tests were scanned with the GE Advance PET tomograph. The crystal sizes for the three tomographs are 4.0 × 8.1 × 30 mm, 6.3 × 4.7 × 30 mm and 2.1 × 2.1 × 10-10 mm (dual layer) respectively. The corresponding FWHM values in the transaxial and axial directions were 5.0 mm and 6.5 mm for the GE Advance, 5.0 mm and 5.8 mm for the PET-CT and 2.5 mm and 2.5 mm for the HRRT tomograph. All FWHM values were acquired according to the NEMA NU-02 standard.[17] Although FWHM degrades when moving out from the center of the field view, in this work it was assumed that the PSF is constant. All radioactivity concentration values were calculated from values measured with a calibration dose calibrator (VDC-404, Veenstra Instrumenten, The Netherlands), the known distribution volume and the time difference between measurement and scan times.

### Phantom data

**Figure 1:**
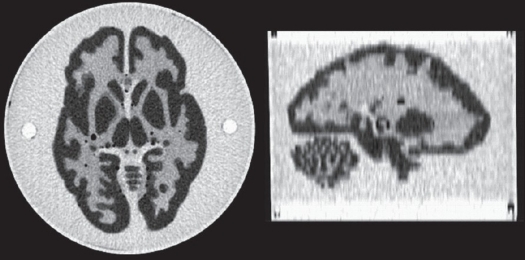
Hoffman phantom: CT image of the CT-PET tomography. A transaxial slice on the left and a sagittal slice on the right.

The accuracy of the quantification was tested using a Hoffman brain phantom (model JB003) that resembles a human brain with respect to the distribution of volumes identified as WM, GM and cerebrospinal fluid (CSF) [Figure 1] The phantom consists of 40 plastic plates of 3 mm thickness, where the GM areas are fully excavated and the WM areas are formed so that they have empty spaces of one-fourth of the plate thickness. The phantom was filled with ^18^F-labeled FDG liquid to follow the 4:1 relation between the GM and WM activities. The used PET image dimensions for the GE Advance, PET-CT and HRRT-PET tomographs were 256 × 256 × 35, 256 × 256 × 47 and 256 × 256 × 207 voxels respectively. For HRRT, the central 100 planes were used in order to reduce computational requirements. With HRRT, 3.0 mm Gaussian post-smoothing was applied in order to avoid the influence of the structure of the Hoffman phantom. The voxel sizes for the tomographs were 1.17 × 1.17 × 4.25 mm, 1.82 × 1.82 × 3.27 mm and 1.22 × 1.22 × 1.22 mm respectively.

To obtain structural information for the phantom studies, a CT image with 512 × 512 × 47 voxels of size 0.59 × 0.59 × 3.27 mm was used [Figure 1]. The usage of a CT image instead of MRI was not expected to produce differences in results, since only the segmentation information is used from these images. Because no mathematical model of the phantom was available, the CT image was segmented to WM and GM regions by first preprocessing it with a median filter and then thresholding it by manually determined low and high threshold values. The resulting CT segment images were already in alignment with the PET-CT PET image, so it was only necessary to re-slice them into the PET image voxel dimensions to give a structural definition of the WM and GM regions. The phantom PET images of the GE Advance and HRRT tomographs were naturally not in alignment with the CT image. These two PET images were co-registered to the PET-CT orientation in order to preserve the segmentation accuracy, which has been reported to be one of the main error factors in PVC with the methods under evaluation.[11] In co-registration, the SPM2 co-registration was used with normalized mutual information (NMI) as a cost function. In the re-slice operation for the PET images, the linear interpolation method was used to calculate the PET image voxel values in the new coordinates. As the bio-distribution is the same and there is no motion during scans, the resulting alignments between CT and PET images were assumed to be uniform.

### Test-retest data

To assess the impact of different PVC approaches on the radioactivity estimates and reproducibility and reliability of [^11^C] raclopride binding in the human brain, we re-analyzed a previously published data set[18]. In brief, eight healthy volunteers had undergone two successive scans with a GE Advance PET tomograph during the same day, at least 2 h apart. All subjects had also undergone 1.5T MR imaging. The image dimensions were 128 × 128 × 35 voxels for the PET and 256 × 256 × 100 voxels for the MRI. Voxel sizes for the PET and the MRI were 2.34 × 2.34 × 4.25 mm and 1.09 × 1.09 × 1.00 mm respectively.

The MR images were segmented by using the segmentation module of the SPM2 software package, which uses a priori probability maps of the GM and WM regions in order to assign voxels in brain space to these segments. The PET images were realigned as previously described,[18] except that SPM2 routines were applied. The ROIs were manually delineated on MR images co-registered to summated PET images using the normalized mutual information method as implemented in SPM2. ROIs were drawn on axial slices on caudate, putamen, lateral and medial thalamus and cerebellum. All ROIs were created using the Imadeus software (version 1.2, Forima Inc., Turku, Finland). The Simplified Reference Tissue Model (SRTM)[19] using the cerebellum as a reference region was applied to the regional time-radioactivity curves to yield binding potential (BP) values in the ROIs, indicating the capacities of receptors to bind the used ligand [^11^C] raclopride. The cerebellum reference region was delineated on an area that was defined as the GM region.

Test-retest reproducibility and reliability of the BP values were assessed using test-retest variability (VAR) and intra-class correlation coefficients (ICC):

$\mathrm{VAR}=\frac{\left|\mathrm{scan2}-\mathrm{scan1}\right|}{0.5x|\mathrm{scan1}\;\mathrm{scan2}|}$

where *scan*1 and *scan*2 refer to BP values of ROI in test and retest scans, and

ICC = BSMS - WSMSBSMS + (n + 1) × WSMS

where *BSMS* is the between-subject mean square of the BP values, *WSMS* is the within-subject mean square and *n* is the number of repeated observations (in this case, *n* = 2). ICC values can range between −1 and 1; values close to 1 indicate that most variance is due to between-subject rather than within-subject variation (good reliability), whereas values below zero imply greater within-subject than between-subject variation (poor reliability).

The mean BP values between test-retest data over eight subjects were compared with t-test (*p* < 0.05, *df* = 7) with noncorrected data and with each of the four evaluated PVC methods. Also, the mean BP values over all 16 studies and the mean variability over eight subjects of noncorrected data were tested to the corresponding values of the four PVC approaches with t-test (*p* < 0.05, *df* = 15 and *p* < 0.05, *df* = 7 respectively). All the t-tests were paired and the t-values were compared to two-tailed t-distribution critical values.

### Partial volume correction

Partial volume correction software PVE Out (http://pveout.area.na.cnr.it) was used for the method evaluation. The system incorporates the methods M-PVC, MG-PVC, R-PVC and A-PVC. The MG-PVC and A-PVC methods utilize WM mean value estimation with the A-WME, R-WME or CS method. As a result, eight PVC methods were available. All the methods were evaluated with a phantom study. The FWHM specified above was used for tomograph-specific PSF information in PVE Out. No brain regions other than the GM and WM were assumed to contribute to the radioactivity distribution. The PSF values used with the test-retest data were the same as those used in the phantom study. To save computational resources, the creation of segmented MR images that were aligned to the PET images was carried out only once for each subject and then applied to each of the correction methods. The PVC processes for phantom and test-retest data were all run as batch processes, which required no user interaction after setting the PSF values and input images. All batch processes were executed with Matlab 6.5 on Windows XP environment with a laptop computer with a 1.80 GHz processor. The MG-PVC and A-PVC methods apply correction only to the GM region, setting other voxel values to zero. Therefore, voxels outside the GM region were removed from the test-retest ROIs to make the results comparable with each other. The co-registration process was carried out once for each PET-MRI image pair and it took about 5 min for each of them. The batch process for each of the three co-registered phantom object image pairs took approximately 10 min. For test-retest data, it took about 25 min for each correction method to process all 16 PET studies.

## Results and Discussion

### Quantification results

Quantification results of the Hoffman phantom images are shown in Table 1. The results are given both before and after the correction for the PET-CT, GE Advance and HRRT-PET tomographs. The PVE-corrected values were divided by the calculated theoretical values to quantify the differences. The difference in mean standard deviation of voxel radioactivity values is also shown in the table.

**Table 1:** Results of partial volume effect correction for a Hoffman phantom

*GM*			*WM*			*PVC method*	*WM estimation*
			
*Bq/ml*	*Recovery rate %*	*SD increase %*	*Bq/ml*	*Recovery rate %*	*SD increase %*
*PET-CT*							
36378	65.3		19863	142.6		Non-corrected	
55067	98.9	37.5	12871	92.4		A-PVC	A-WME
52414	94.1	49.4	16600	119.2		A-PVC	R-WME
51818	93.0	57.4	17438	125.2		A-PVC	CS
50231	90.2	21.6	12871	92.4		MG-PVC	A-WME
49628	89.1	21.5	16600	119.2		MG-PVC	A-WME
49492	88.9	21.6	17438	125.2		MG-PVC	CS
46177	82.9	20.4	21854	156.9	24.3	M-PVC	
52154	93.6		16603	119.2		R-PVC	
*GE advance*							
11266	60.6		6625	142.5		Non-corrected	
17602	94.6	56.1	4345	93.4		A-PVC	A-WME
16682	89.7	68.7	5629	121.0		A-PVC	R-WME
17505	94.1	54.6	4481	96.4		A-PVC	CS
15815	85.0	30.8	4345	93.4		MG-PVC	A-WME
15595	83.8	29.8	5629	121.0		MG-PVC	A-WME
15792	84.9	30.6	4481	96.4		MG-PVC	CS
11492	77.9	29.3	7296	156.9	25.1	M-PVC	
16016	86.1		5907	121.0		R-PVC	
*HRRT*							
37205	80.0		16084	138.4		Non-corrected	
49254	105.9	48.9	11302	97.2		A-PVC	A-WME
47322	101.8	55.4	13633	117.3		A-PVC	R-WME
48405	104.1	44.6	12326	106.0		A-PVC	CS
44283	95.2	13.0	11302	97.2		MG-PVC	A-WME
43989	94.6	12.7	13633	117.3		MG-PVC	A-WME
44154	95.0	12.8	12326	106.0		MG-PVC	CS
41506	89.3	16.6	16222	139.5	2.2	M-PVC	
45429	97.7		13633	117.3		R-PVC

The mean radioactivity values (Bq/ml) are shown for areas of GM and WM for three different PET tomographs: GE Advance (GE, Milwaukee, WI), PET-CT (DSTE, GE, Milwaukee, WI) and HRRT (CPS Inc., Knoxville, TN)). Recovery rate = Radioactivity in relation to the calculated true values of GM and WM; SD increase = Increase in standard deviation of the radioactivity values in the GM and WM regions when compared to noncorrected image when applicable.

*PVE-corrected mean radioactivity:* The main focus of the evaluated PVC methods is to correct the PET GM radioactivity values. The corrected WM values are used when calculating the PVE-corrected GM activities, except in the case of the R-PVC method, which gives equal focus to all the regions.

The relative performance of the three WM estimation methods (A-WME, R-WME, CS) varied depending on the PET tomograph. The R-WME estimates were higher than the A-WME estimates with all the three PET tomographs. With the GE Advance and HRRT, the CS method gave good estimates for WM; but with the PET-CT, considerable overestimation occurred. The PVE-corrected WM mean value for the M-PVC method was higher than the noncorrected value in all the tomographs. The MG-PVC method was more robust to the changes of the WM estimation value than the A-PVC method, as seen from the GM values when using different WM estimations in Table 1.

In the corrected GM radioactivity values, there was a general trend that the correction efficiency decreased in the following order of PVC methods: A-PVC, R-PVC, MG-PVC and M-PVC. The highest efficacy was observed for the A-PVC method. The same order of results held generally for all three tomographs. The GM activity calculated for the HRRT tomograph was relatively good, even prior to PVC. Due to having the highest tomograph resolution, the PVE-corrected mean radioactivity values with HRRT were also closest to the calculated values. The HRRT tomograph with A-PVC slightly overestimated the mean activity, but the percentage error was still of the same magnitude as with R-PVC and MG-PVC.

**Figure 2:**
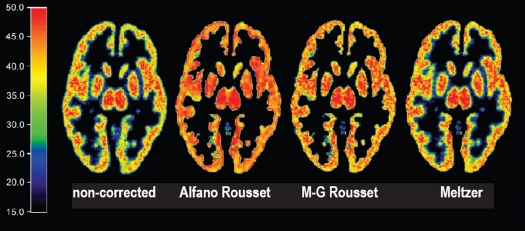
Distribution of the standard deviation in a PET image before and after PVC (Bq/ml). Standard deviation images from left or right: noncorrected image, Alfano *et al*., Müller-Gärtner *et al*., Meltzer *et al*. WM estimation by Rousset *et al*. was used in correction of the two methods in the middle.

*Distribution of the radioactivity values:* The PVC not only changes the mean radioactivity values of the regions but also affects their distributions [Figure 2], when a 3 × 3 × 3 voxel kernel was used to calculate the standard deviation for each voxel. With the MG-PVC method, the increase in standard deviation of voxel values in GM and WM varied between the PET tomographs, but it produced a small increase in deviation with all the tomographs [Table 1]. It was not possible to calculate the change in standard deviation in the WM region for the A-PVC and MG-PVC methods, since these methods produce only an estimate of the true mean radioactivity value for that region. The R-PVC method did not provide standard deviation for either GM or WM.

### Test-retest results

The mean BP values and mean variability between test sets were calculated for the four regions of interest. In addition, the ICC values were calculated for the eight PVC methods. The values were acquired by correcting the image data from eight human subjects scanned twice. A summary of four of the methods is shown in Table 2. The results of the A-PVC and MG-PVC methods used with the WM estimation methods other than A-WME were essentially similar. As a general observation, almost all the tested correction methods increased the mean BP values in all the examined brain ROIs. A-PVC gave higher BP values than MG-PVC in almost all cases. This result is in accordance with the phantom tests. The R-PVC method resulted in mean BP estimates of [^11^C] raclopride close to the mean values of A-PVC and MG-PVC. The mean BP values were lowest with the M-PVC method. The mean BP values of the methods varied most in the thalamus regions, which were particularly difficult to segment. The standard deviation of the calculated BP increased as a result of PVC in most of the ROIs.

**Table 2:** Effect of the positron emission tomography PVC methods on binding potential estimated by simplified reference tissue model for different brain regions

		*Brain region of interest*			
		
*Method*		*Caudate*	*Putamen*	*Lateral thalamus*	*Medial thalamus*
	Mean ± SD	1.81 ± 1.63	1.31 ± 1.22	2.10 ± 1.69	1.82 ± 1.58
No correction	VAR	3.45	4.93	7.88	8.25
	ICC	0.98	0.91	0.83	0.89
	t-value	0.69	1.03	0.60	1.23
	Mean ± SD	2.82 ± 2.60	2.27 ± 1.81	2.71 ± 2.50	2.04 ± 1.84
Alfano *et al*.	VAR	5.49	5.84	4.76	6.83
WME Alfano	ICC	0.92	0.94	0.98	0.75
	t-value	0.76	1.16	1.24	1.22
	Mean ± SD	2.50 ± 2.34	1.86 ± 1.70	2.40 ± 2.26	2.31 ± 1.88
Müller-Gärtner *et al*.	VAR	4.35	6.13	6.32	8.23
WME Alfano	ICC	0.94	0.88	0.93	0.77
	t-value	0.76	1.02	0.85	1.48
	Mean ± SD	2.03 ± 2.39	1.28 ± 1.60	2.24 ± 2.30	1.70 ± 1.90
Rousset *et al*.	VAR	4.08	5.92	8.89	11.41
no WME	ICC	0.97	0.92	0.94	0.84
	t-value	2.20	1.98	0.81	1.06
	Mean ± SD	1.92 ± 1.68	1.33 ± 1.24	2.19 ± 1.71	1.84 ± 1.58
Meltzer *et al*.	VAR	3.25	5.21	7.99	9.15
no WME	ICC	0.98	0.92	0.87	0.91
	t-value	1.65	1.15	0.86	1.31

The test set consisted of eight subjects. Each subject was scanned twice. Mean = Mean value of BP over all 16 scans; VAR = Mean variability between two scans over eight subjects; t-value = t value between the mean values of test and retest scans (df = 7, critical value = 2.306 with *P* < 0.05); ICC = Mean intraclass correlation value between scabs over eight subjects.  Alfano *et al*., and Müller-Gärtner methods were used with WM estimation method by Alfano *et al*. as no difference was seen between WM corrections.

The variation in the ICC values was one of the main issues of interest, and there were indeed large differences between the different methods in this respect. The M-PVC method gave ICC values that were greater than or similar to the noncorrected images in all ROIs. The R-PVC method also performed well, with a slight decrease in the results of the caudate and medial thalamus regions.

The performance of the A-PVC and MG-PVC methods varied among the examined regions. In the lateral thalamus region, all the PVC ICC values were greater than the noncorrected values. In the medial thalamus, the A-PVC and MG-PVC methods resulted in markedly smaller ICC values than with noncorrected images. The null hypothesis in the test-retest data was that the mean BP values of the test and retest scans would be equal. This hypothesis was not disproved in any region with any PVC method (*þ* < 0.05, [Table 2]). The mean BP value over all 16 studies with noncorrected data was found to be significantly different from the corresponding values of all PVC methods (*þ* < 0.05) except in the putamen and lateral thalamus regions with M-PVC (in putamen, significance found when *P* < 0.1). For the mean variability (VAR) over eight subjects, the only significant differences were found with A-PVC in the caudate region (*P* < 0.05, variability increased) and in the thalamus region (*P* < 0.1, variability decreased).

## Discussion

The main purpose of PVC is to improve the quantitative characteristics of PET images. The effect of PVC for a single scanning session was tested with a phantom object, with the focus on the mean values of ROIs before and after the PVC. The results of these experiments were generally as expected concerning the increase in radioactivity values; the difference in ROI mean values before and after the correction operation follows the results reported earlier.[10,11,16] The situation deviates strongly from the above when the interest is especially on PVE-corrected images.[20] While the voxel-based PVC methods provide more possibilities for further PET analyses, they are not very robust to noise. As can be seen from the results of the phantom tests [Table 1 and Figure 2], the standard deviation of voxel values increases with the mean radioactivity values. This affects the calculation of the [^11^C] raclopride BP in the present findings. With the test-retest data on humans, the M-PVC method was the only method which did not decrease the ICC values in any tested region.

The main observation was that it is difficult to correct PVE with high radioactivity restoration while at the same time keeping the standard deviation low. No single method performed better than the others in all aspects. To choose the most suitable PVC method, a compromise must be made between radioactivity restoration and the reproducibility and reliability of PET scans. Some issues related to choosing the PVC method are discussed in the following in more detail.

## Noise amplification

It is a trivial finding that the corrected voxel values of a PET image increase rapidly when the divider approaches zero in formulae (2-4), (2-6), (2-9). The division by the convoluted mask image is done in M-PVC, MG-PVC and A-PVC, and the effect is largest at the edges of the target region. In the center of the region, the divisor is 1 because all radioactivity is supposed to come only from the same region even after smoothing with the PSF. This phenomenon explains the differences in noise amplification between M-PVC, MG-PVC and A-PVC. The M-PVC method amplifies noise at the edges of the conjunction of the GM and WM regions, whereas MG-PVC and A-PVC also amplify noise in the border area between the GM and WM. This effect is clearly visible in Figure 1 when comparing MG-PVC and M-PVC.

Since in the reproducibility tests, all the defined regions were located near the border area of WM and GM, the noise amplification has a considerable effect on all these regions for MG-PVC and A-PVC. This is clearly observable in the medial thalamus region, where only M-PVC gave satisfactory results. The thalamus area seems to be, in general, difficult to segment, and this may have increased the variability of the results in this region. Noise amplification due to PVC need not be problematic when considering the mean radioactivity values in a single scanning session.

However, the effect might be noticeable if further voxel-based analyses are performed. For MG-PVC, the noise amplification was approximately of the same magnitude as for M-PVC when applied to all three tomographs. In addition, MG-PVC had good radioactivity value restoration, which suggests that this method might be a good candidate for further voxel-based analyses in single-scan studies.

## Accuracy of ROI mean values

For PET studies in which each subject is scanned only once, it can be more important to have accurate radioactivity values than to have additional reliability in the studies when the interest is in actual value magnitudes rather than in changes between conditions of multiple scans. In the phantom tests of the present study, the true mean radioactivity magnitude restoration was smaller with M-PVC than with the other three methods. Moreover, the t-test between the mean BP values in the caudate and lateral thalamus regions with M-PVC did not indicate significant difference from noncorrected BP values. We therefore draw the following conclusions for A-PVC, R-PVC and MG-PVC:

- A-PVC produced the best mean radioactivity restoration; hence this method could be used when one is looking for PVE-corrected mean radioactivity values without further voxel-based image analysis or reproducibility.
- R-PVC gave weaker radioactivity restoration than A-PVC for the phantom. With the GE Advance PET, it was seen to produce uniform ICC values between the studied regions. This method could be useful when only the mean radioactivity value of the region is used and both radioactivity value restoration and reproducibility are needed.
- MG-PVC could be considered a compromise between A-PVC, which is quantitatively accurate; and M-PVC, which better preserves the voxel characteristics in ROIs. MG-PVC is robust to errors in WM estimation, so either R-WME or A-WME could be used as its WM estimator.

Important issues in the calculation of the ROI mean value by A-PVC or MG-PVC are the volume of ROI and its vicinity to the GM border. The former affects the mean sensitivity; and the latter, the increases in local radioactivity variation due to the noise amplification in PVC. Because ROIs often tend to lie at the border of GM, this becomes an issue. On the other hand, when ROIs contain voxels from WM, the estimation of the radioactivity values of WM voxels may crucially affect the usableness of A-PVC and MG-PVC. In order to include the WM voxels in an ROI, the estimated WM mean value may be used as corrected values of the WM voxels. Another alternative is to exclude the noncorrected voxels of ROIs when the number of remaining voxels is reasonable, as was the case in the present study. In contrast, the R-PVC method does not suffer from such a weakness. The M-PVC method solves the issue by not using the border between GM and WM.

## Intra-class correlation

Not only the capability of restoring the mean radioactivity values in ROIs but also the reproducibility and reliability are essential properties of PET. Its role should be underlined in studies where the same subject is scanned multiple times. Then, if all true PVE were corrected by a PVC method, the ICC values would be at least the same as without correction. If the BP values remain the same with correction as without it, PVC naturally has no effect on ICC values and the observed variance between scans is due to other factors, such as biological variability.

When ICC values decrease as a result of PVC, the reason might be that the correction has added additional variability between the scans. On the basis of the results of the test-retest experiment [Table 2], M-PVC is more robust as regards the ICC values; the region of the medial thalamus posed a challenge to all other PVC methods.

When the image intensity values approach some constant value, the characteristic numbers of the reproducibility test automatically approach their optimal values (std and variability tend toward zero and ICC toward 100%). Because of this, quantification tests should be carried out in conjunction with the reproducibility test. From the phantom test [Table 1], it was observed that the M-PVC method overestimates the radioactivity in the WM region and this can affect the ICC values of the thalamus regions. However, the effect is expected to be small, especially for the medial thalamus since it is located mainly in the GM region. Therefore, on the basis of the experiment, we conclude that the M-PVC method is the preferred choice for real human studies in which individual subjects are scanned repeatedly. This conclusion, however, includes the assumption that less radioactivity value restoration is acceptable.

## Conclusion

Four PVC methods (M-PVC, MG-PVC, R-PVC, A-PVC) and three WM estimation methods (CS, A-WME, R-WME) were tested in order to evaluate their performance in studies of WM/GM radioactivity measurements. The methods were tested with the Hoffman phantom for three PET tomographs (GE Advance, GE DSTE and Siemens HRRT), to address the performance of the methods with tomographs of different features. Of these, the tomograph resolution can be considered as the factor with the greatest consequence on the magnitude of partial volume effects. The correction methods were also tested with test-retest data taken from two different scans of eight subjects, totaling 16 PET images. Variability and ICC values were calculated from the binding potential values of the test-retest sets with and without PVC. Our main observation was that the sophisticated A-PVC method most improved the PET accuracy, which is useful when activity values of ROIs are compared between study groups. Although M-PVC had weaker single-study accuracy, it improved or maintained tomograph reliability even in regions where other methods failed (caudate, putamen, medial thalamus). This property is beneficial in test-retest study designs such as drug-effect studies. The choice between these two methods should finally be made on the basis of the research question of the study.
